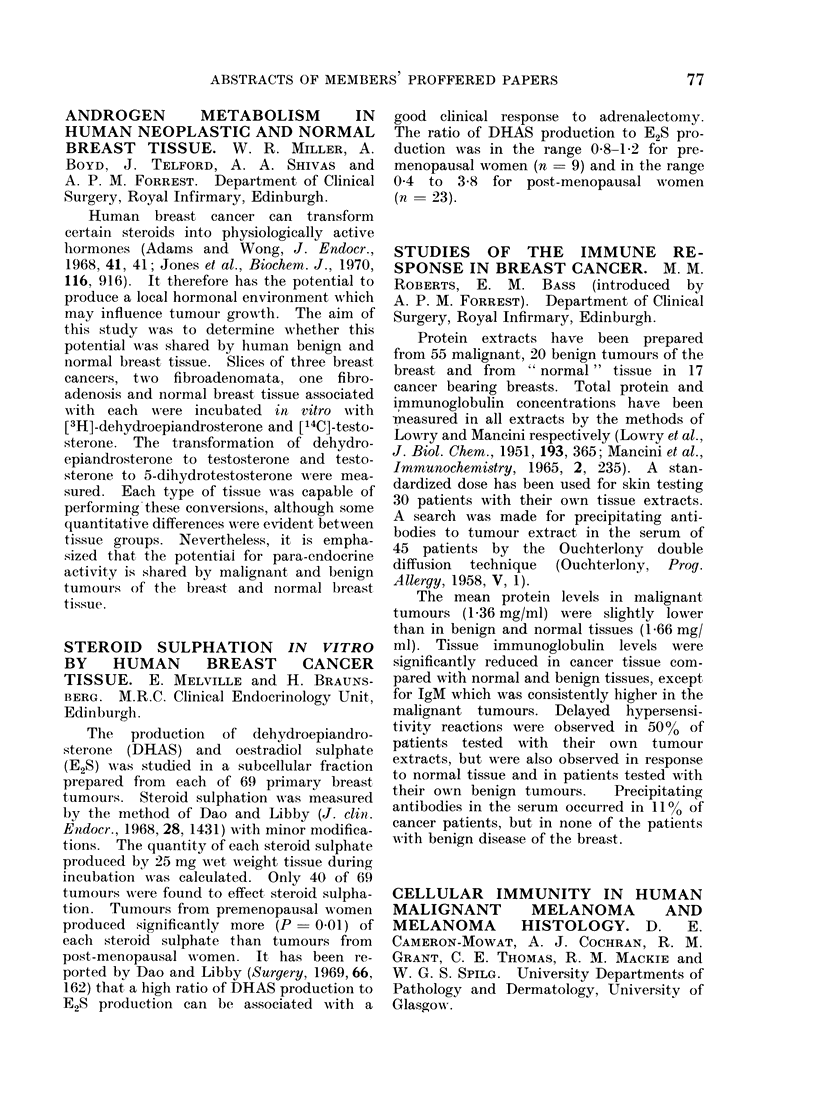# Studies of the immune response in breast cancer.

**DOI:** 10.1038/bjc.1973.78

**Published:** 1973-07

**Authors:** M. M. Roberts, E. M. Bass


**STUDIES OF THE IMMUNE RESPONSE IN BREAST CANCER.** M. M. ROBERTS, E. M. BASS (introduced by A. P. M. FORREST). Department of Clinical Surgery, Royal Infirmary, Edinburgh.

Protein extracts have been prepared from 55 malignant, 20 benign tumours of the breast and from "normal" tissue in 17 cancer bearing breasts. Total protein and immunoglobulin concentrations have been measured in all extracts by the methods of Lowry and Mancini respectively (Lowry *et al.*, *J. Biol. Chem.*, 1951, **193**, 365; Mancini *et al.*, *Immunochemistry*, 1965, **2**, 235). A standardized dose has been used for skin testing 30 patients with their own tissue extracts. A search was made for precipitating antibodies to tumour extract in the serum of 45 patients by the Ouchterlony double diffusion technique (Ouchterlony, *Prog. Allergy*, 1958, **V**, 1).

The mean protein levels in malignant tumours (1·36 mg/ml) were slightly lower than in benign and normal tissues (1·66 mg/ml). Tissue immunoglobulin levels were significantly reduced in cancer tissue compared with normal and benign tissues, except for IgM which was consistently higher in the malignant tumours. Delayed hypersensitivity reactions were observed in 50% of patients tested with their own tumour extracts, but were also observed in response to normal tissue and in patients tested with their own benign tumours. Precipitating antibodies in the serum occurred in 11% of cancer patients, but in none of the patients with benign disease of the breast.